# Development and Analytical Evaluation of a Microarray Assay for Quantitative Determination of Human Blood IgG Reactive to Food Antigens in the Italian Population

**DOI:** 10.17113/ftb.64.02.26.8892

**Published:** 2026-06-15

**Authors:** Veronica Mantovani, Federica Capitani, Francesca Maccari, Fabio Galeotti, Nicola Volpi

**Affiliations:** Department of Life Sciences, Laboratory of Biochemistry and Glycobiology, University of Modena and Reggio Emilia (Unimore), Modena, Italy

**Keywords:** microarray, IgG evaluation, food antigens, food intolerance, food hypersensitivity

## Abstract

**Research background:**

Food intolerance mediated by IgG antibodies is an adverse reaction resulting from the difficulty to digest or metabolize a food or food component(s) and is manifested by numerous nonspecific symptoms, potentially also provoking systemic inflammation and allergy symptoms. Consequently, the detection of circulating food-specific IgG has a diagnostic value with possible clinical applications.

**Experimental approach:**

We produced microarray chips able to analyse 16 blood samples simultaneously. After validation of the characteristics and performance using stringent quality control criteria, we investigated their diagnostic validity for IgG-mediated intolerance in 6575 subjects from the Italian population to 92 purified food proteins. Moreover, this assay was performed on capillary blood samples collected from the fingertip, permitting a minimally invasive and practical collection of blood.

**Results and conclusions:**

Sixteen antigens showed an IgG response greater than 10 % and eight aliments of these had a reactivity greater than 15 %. Wheat (20.4 %), cow’s milk (30.8 %), brewer's yeast (23.4 %) and mozzarella cheese (22.4 %) produced a very high IgG response, greater than 20 %, probably due to their widespread use in the Italian cuisine. Goat’s milk, various milk derivatives (like gorgonzola, Grana Padano and parmesan cheese), durum wheat, kamut, egg and gluten had a reactivity greater than 15 %. Four food antigens (emmer, pecorino cheese, ricotta and rye) caused a moderate IgG reactivity ranging between 10 and 15 %. The other food extracts showed a mean low IgG reactivity below 10 %. No significant differences were observed between female and male subjects or among the various Italian regions analysed. In contrast, the highly reactive food antigens showed a decrease in IgG response with increasing age.

**Novelty and scientific contribution:**

After validation, our microarray chips proved to be a robust method with good reproducibility and low variation. Even if the primary aim of this study is to evaluate the incidence of IgG-mediated reactivity in the Italian population using a novel microarray technology and to compare the results with a previous study conducted using enzyme-linked immunosorbent assay **(**ELISA), this analytical approach can also help identify the triggers of intolerance symptoms and help doctors or nutritionists in selecting the best treatment for patients, with the additional aim of clarifying the controversy surrounding IgG testing. Finally, our microarray technology enables high throughput, ensuring that a large number of samples can be analysed with significant savings in time, reagent and costs, while remaining minimally invasive for patients.

## INTRODUCTION

Food allergy is a reaction of the immune system to a food or its component(s) producing higher levels of IgE reactive to that food or its protein(s) (*1*). The symptoms of allergy appear shortly after consuming the food, generally within 2–4 h, and are more severe the earlier they occur. An estimated 6-8 % of children (*1*) and almost 6 % of adults in Europe (*2*) and the United States (*3*) suffer from food allergies, including multiple allergies such as to milk, eggs and peanuts simultaneously (*1*-*3*). Food allergy significantly affects the quality of life of patients and their families, with significant health care costs for individuals and National Health Systems. Consequently, since 2014, European Regulation 1169/2011 has been enforced in Italy (*4*) which makes allergen reporting mandatory even for non-prepacked foods. Food allergy is defined as a specific and reproducible immunological reaction related to food ingestion.

In contrast to food allergy, food intolerance is an adverse reaction resulting from the body's inability to digest or metabolize a food or food component (*5*) and is manifested by numerous nonspecific symptoms. The most commonly reported food intolerances causing gastrointestinal symptoms (pain, abdominal distension, flatulence, and diarrhoea) are wheat bread, milk and related products, and spiced foods (*6*). Food intolerance, mediated by IgG antibodies (*6*), can promote systemic inflammation and has been associated with allergy symptoms such as rashes, urticaria and asthma, but the pattern of the most common food-specific IgG frequently observed may vary among different populations.

The detection of circulating food-specific IgG has a diagnostic value as it has been performed and associated with several pathological and clinical conditions, such as subjects with small bowel stomas (*7*), those affected by migraine and its comorbidities (*8*), autism spectrum disorder (*9*), inflammatory bowel disease (*10*), ulcerative colitis (*11*), childhood eczema (*12*), Crohn's disease (*13*), asthma (*14*) and, in general, with allergic symptoms (*5*, *6*).

Apart from some techniques and procedures without any scientific basis, such as cytotoxic food testing, the antigen leukocyte antibody test (ALCAT), bioresonance, electrodermal testing (electroacupuncture), reflexology and applied kinesiology (*15*, *16*), the diagnosis of IgG-mediated food intolerances is based on scientifically sound tests known as enzyme-linked immunosorbent assay (ELISA). These are used for the evaluation of serum immunoglobulin responses in normal and pathological subjects, performed on 96-well plastic supports or on chips belonging to the latest investigative technique, known as microarray technology (*17*). Microarrays originated as useful tools for genotyping and gene sequencing, with clinical applications as both diagnostic and prognostic tools (*17*). In addition to these uses, microarrays are also used in nutrigenomics to study individual responses based on genetic makeup (*18*). More recently, microarrays have been used to detect IgE in rapid serological tests (*18*) and applied for the evaluation of possible allergens, as well as food and environmental allergies (*19*).

In our previous study (*20*), we used the ELISA test to quantify the IgG in the serum of 6879 subjects from the Italian population reactive to 160 different food extracts. In the present study, we produced microarray chips and, after validating their characteristics and performance by stringent quality control criteria, we investigated their diagnostic validity for intolerance of 6575 subjects of the Italian population to 92 purified food proteins. Moreover, we performed this assay on capillary blood samples collected *via* fingertip using an appropriate swab according to our patent (*21*), permitting minimally invasive and practical blood collection. This study was conducted blindly, with the primary aim of evaluating the incidence of IgG-mediated reactivity in the Italian population using our microarray technology, as described here, and comparing the results with the previous study using ELISA (*20*). The condition of the enrolled subjects, including any possible pathologies or symptoms related to clinically relevant food intolerances, was unknown. A second study is currently underway to correlate possible pathologies with IgG-mediated reactivity to food-derived proteins.

## MATERIALS AND METHODS

### Materials

For the analytical procedure, porous nitrocellulose film microarray slides (Grace Bio-Labs, Bend, OR, USA) were used. Technical grade tris(hydroxymethyl)-aminomethane (TRIS), NaCl, Proclin 300, Tween® 20 and 3,3’,5,5’-tetramethylbenzidine (TMB) were obtained from Sigma-Aldrich Chemie GmbH, Merck (Schnelldorf, Germany). Protease inhibitor cocktail tablets were from Roche Diagnostics GmbH (Mannheim, Germany). Human IgG from serum and goat-produced peroxidase (HRP)-conjugated goat anti-human IgG (Fab specific) were from Invitrogen (Waltham, MA, USA). Bovine serum albumin (BSA) was from NZYtech (Lisbon, Portugal). ELISA 96-well plates from Greiner Bio-One (Cassina de Pecchi, Milano, Italy) were also used for sample preparation.

### Production of food antigens

Food antigens were produced from 92 food items (Table S1) using a proprietary extraction procedure. Briefly, all food items were diluted in a buffer at pH=7.5 containing 50 mM TRIS, 120 mM NaCl, 0.1 % Tween 20, 0.1 % BSA and a protease inhibitor cocktail. The solutions were homogenised in an Ultra-Turrax (Kinematica AG, Malters, Switzerland) and the homogeneous food mixtures were transferred into previously labelled tubes, vortexed, incubated for 10 min at -20 °C and subjected to cold agitation for 30 min. After an extraction for approx. 2 h, the food mixtures were centrifuged (SCI24 high speed micro-centrifuge; SCILOGEX LLC, Rocky Hill, CT, USA) at 5000×*g* for 15 min, and the supernatants were then collected into pre-labelled tubes and incubated overnight at 4 °C. The food protein extracts were then centrifuged at 20 000×*g* for 15 min at 10 °C and the supernatants were diluted in 50 % glycerol and frozen at -20 °C.

The extraction of food proteins was quantitatively evaluated by a Bradford spectrophotometric assay (*20*) and qualitatively by sodium dodecyl sulfate-polyacrylamide gel electrophoresis (SDS-PAGE) (*20*).

### Human subjects

For this study, 6575 subjects aged approx. 1 to 93 years were selected from the Italian population: 1258 males, 3106 females and 2211 of unknown sex. The mean age of the males was 41±16 years, while that of the females was (42±17) years.

Biological samples were provided by a private laboratory (Unifarco SpA, Belluno, Italy). The samples were anonymized and sent to a second laboratory (Diagnostica Spire, Reggio Emilia, Italy), which carried out the IgG-mediated reactivity analyses. After the analyses, the non-identifiable aggregated data were collected and used in this research.

### IgG microarray

Food antigen extracts were diluted in a buffer at pH=7.5 containing 50 mM TRIS, 120 mM NaCl, 0.1 % Tween 20 and 0.1 % BSA. The diluted food antigen extracts were spotted using the Spotter iONE M24YOU (Berlin, Germany) on porous nitrocellulose film microarray slides, which were then stored at 4 °C overnight.

The microarray slides were subsequently used to analyse the capillary blood samples by a colorimetric assay based on ELISA to determine IgG immunoglobulins reactive to food antigens. Briefly, the microarray slides were immersed in a blocking solution to cover non-specific sites for 30 min. After the blocking phase, the diluted capillary blood samples were added to each microarray slide pad and incubated for 30 min. After washing with an appropriate buffer, anti-human IgG conjugated with horseradish peroxidase was added. The unbound conjugate was then removed by washing with an appropriate buffer. Finally, a solution containing the peroxidase enzyme (TMB) was added until the completion of the colorimetric enzymatic reaction, which was stopped with a final washing in water.

As it is well known that a single subclass of IgG is not solely responsible for food allergy, the reactivity of food antigens was tested against the entire class of IgG (see Results and Discussion section).

### Statistical analysis

Data are expressed as mean value±coefficient of variation (CV/%). Statistical analysis was performed using analysis of variance (ANOVA) and Student-Newman-Keuls test when appropriate, with IBM SPSS Statistics software, v. 19.0 (IBM, Armonk, NY, USA), according to Coenen *et al.* (*22*). Statistical significance was set at p<0.05.

## RESULTS AND DISCUSSION

### Food antigens

**Fig. 1:**
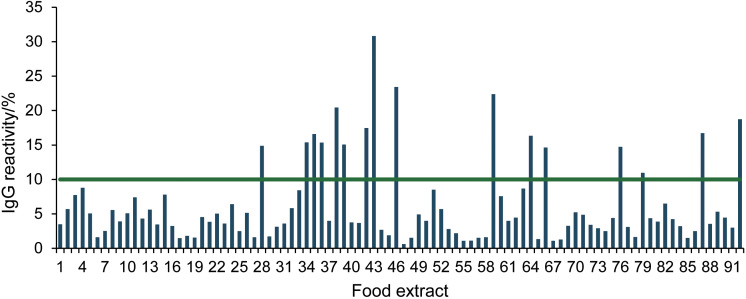
IgG reactivity of the 92 food antigens evaluated by the validated microarray test measured in 6575 subjects from the Italian population. The 92 food extracts are shown in Table S1

According to the previous study (*20*), food antigens were produced from different groups of foods, such as meat, fish, crustaceans, dairy products and eggs, vegetables, seeds, fruits and dried fruits, legumes and cereals, spices, yeast and mushrooms (Table S1). Total proteins from each food were extracted using a proprietary procedure in bulk as previously described. The final proteins were dissolved in a solution containing a cocktail of protein inhibitors and stabilisers. Protein concentration, expressed in mg/mL for each food, serves as a marker for the reproducibility of the extraction and purification protocol used, as well as the concentration suitable for further analysis and microarray spotting. SDS-PAGE and densitometric scanning were performed for each protein extract to determine the relative percentage of each electrophoretic band and the molecular mass of the proteins (see Fig. 1 in (*20*)). These analytical approaches, together with their IgG response, were useful to evaluate the general protein composition of the extracts and the quality control of the production process, as well as to guarantee the reproducibility of the microarray chips.

### Analytical procedure for microarray chips

The current microarray platform is a rapid and high throughput colorimetric test based on the ELISA method to quantitatively measure IgG antibodies to different food antigens, in fresh human serum, plasma, or dried blood. This is not a test for IgE-mediated allergy. IgG antibodies present in dried blood samples were extracted using an appropriate buffer (see above section) and the resulting solutions were tested for reactivity to food antigens. After incubating the microarray slide with the blocking solution, diluted fresh plasma, serum, or extracted dried blood samples were incubated with food antigens (see above section) immobilised on pads. After washing away unbound serum or plasma components, anti-human IgG conjugated to horseradish peroxidase was added, binding to surface-bound antibodies during the second incubation. A washing step is necessary to remove unbound conjugate, and a solution containing enzyme substrate was added to detect specific antibody binding and resulting colour change of the solution. After removing excess substrate solution, slides were dried by centrifugation for 30 s. The absorbance of the standards, positive controls and samples was measured using a high-resolution scanner and its dedicated software. Results are reported as picograms of IgG, calculated using a standard curve of human IgG at different amounts, from 0 to 24 picograms (pg).

According to our analytical procedure, fresh whole or dried blood samples remained stable for 72 h at 4 °C. Fresh blood was centrifuged at 2500×*g* (4000 rpm) to separate plasma or serum, which are then stable for 30 days if stored at -20 °C. Similarly, dried blood extracts are stable at -20 °C for 30 days. Before use, all tested samples were allowed to reach room temperature and were mixed in the diluent solution. Results may be compromised if plasma, serum, or dried blood extract samples are old or not properly stored. In general, repeated freezing and thawing of samples should be avoided.

### Performance and quality control of microarray chips

**Fig. S1:**
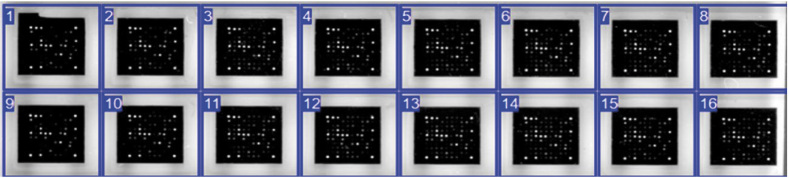
An example of the produced microarray chip consisting of 16 pads, each bearing the 92 food antigens, is shown. Four positive controls are located at the four corners of each pad, with one negative control and increasing concentrations of IgG standard spots visible in the top left corner of each pad

An example of a microarray consisting of 16 nitrocellulose pads is shown in Fig. S1. Each pad on the microarray slide contains 4 positive controls, 1 negative control and spots with increasing masses of IgG standard. A proprietary and patented software integrated with artificial intelligence performs the quality control test for each pad, identifying at least 3 out of 4 positive controls, the negative control, and the linearity of the standard curve (*21*). The linear regression acceptability limit (R^2^) calculated from the calibration curve values of the IgG standards for each pad must be greater than 0.900. Each pad must meet the requirements to pass the test and obtain the final report.

Repeatability was assessed using three serum samples under constant parameters (same operator, measurement system, day and kit lot), obtaining CV values of 12.7, 11.3 and 13.1 %. Reproducibility was assessed using three serum samples under varying parameters (different operator, measurement systems, days and kit lots), with CV values of 12.7, 11.8 and 12.6 %. Repeatability and reproducibility values of CV below approx. 15 % were obtained when dried blood samples were tested instead of fresh serum, both collected from the same human subjects.

The analytical sensitivity of 3.6 pg for plasma, serum, and dried blood extracts was estimated based on the increasing masses of IgG standards used to obtain the standard curve. The linearity of the system, defined as the ability of a method to provide results proportional to the mass of analyte in the test sample within a given range, was assessed using serial dilutions of two different serum and dried blood extract samples with known activity. Mean values of 100.6 % for the first sample and 95.4 % for the second were obtained as percentages of recovery. Accuracy was assessed by diluting a serum or dried blood extract with known activity with a serum or dried blood extract with unknown activity, taking into account the dilution factor used. A recovery of 105.0–103.7 % was calculated for the first sample and a recovery of 109.6–106.5 % was obtained for the second.

According to stability tests, microarray slides were stable for 12 months when correctly stored in their vacuum-sealed package at 2–8 °C. Once opened, they remain stable for 2 months if stored at room temperature in a dry place protected from light.

### IgG response to food antigens

Considering the analytical sensitivity of our chip microarray, we set a limit of 5 % (4.60 pg) for very low IgG reaction, 10 % (5.6 pg) for medium reactivity, 15 % (6.60 pg) for moderately high and >20 % (7.60 pg) for very high reactivity antigens.

Fig. 1 shows the IgG reactivity values of the antigens measured in 6575 subjects from the Italian population. As evident, 16 antigens show an IgG response greater than 10 % and 12 of these foods have a reactivity greater than 15 %. Four food extracts produced a very high IgG response, greater than 20 %, in particular wheat (20.4 %), cow’s milk (30.8 %), brewer's yeast (23.4 %) and mozzarella cheese (22.4 %), probably due to their widespread use in Italian cuisine. The other food extracts show a mean low IgG reactivity below 10 %. It is worth mentioning that these are mean values determined from a very large representative sample of Italian population, 6575 subjects, and IgG response ranged from 0 to 100 % for each food antigen studied depending on the individual. Milk (from cow and goat), various milk derivatives (like gorgonzola, grana Padano, Parmesan cheese and mozzarella cheese), soft and durum wheat and kamut, were found to produce a high IgG response, along with egg, yeasts and gluten (Fig. 1). Moreover, 4 food antigens (pecorino cheese, ricotta, emmer and rye) were found to give moderately high IgG reactivity, ranging between 10 and 15 %.

**Fig. 2:**
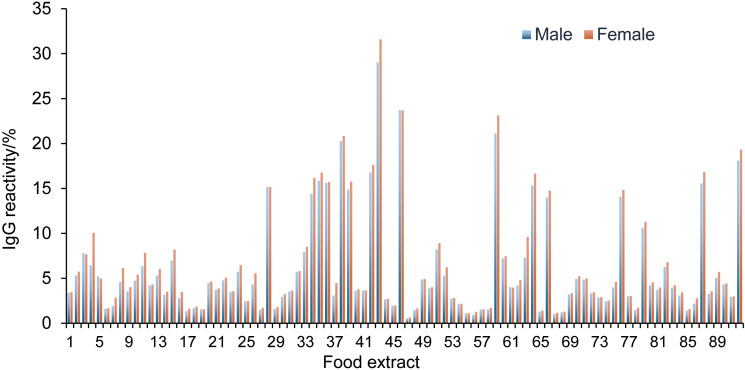
IgG reactivity of the 92 food antigens in females and males from the Italian population. The 92 food extracts are shown in Table S1

**Fig. 3:**
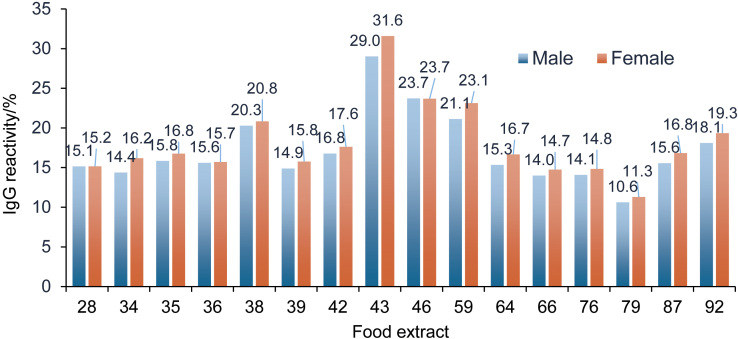
IgG reactivity of 16 highly reactive (>15 %) 92 food antigens in females and males from the Italian population. 28=Emmer cheese, 34=gorgonzola cheese, 35=Grana Padano cheese, 36=durum wheat, 38=wheat, 39=kamut, 42=goat’s milk, 43=cow’s milk, 46=brewer’s yeast, 59=mozzarella cheese, 64=Parmesan cheese, 66=Pecorino cheese, 76=ricotta cheese, 79=rye, 87=egg, 92=gluten

No significant differences were observed between female and male subjects for both low (Fig. 2) and highly reactive (Fig. 3) 92 food antigens studied.

**Fig. 4:**
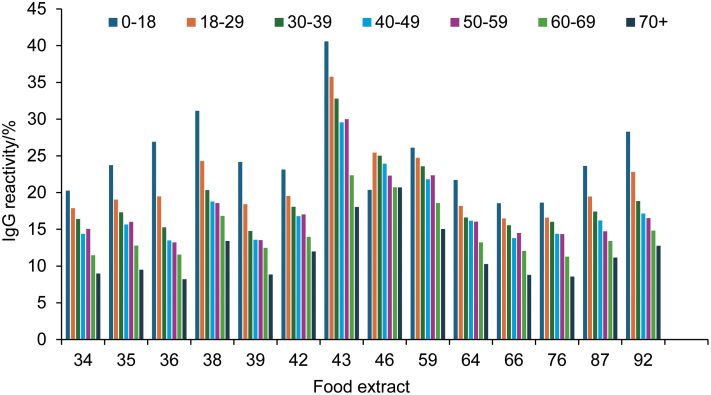
IgG reactivity of 14 highly reactive (>15 %) food antigens in relation to the age of the Italian subjects, divided in seven groups. 34=gorgonzola cheese, 35=Grana Padano cheese, 36=durum wheat, 38=wheat, 39=kamut, 42=goat’s milk, 43=cow’s milk, 46=brewer’s yeast, 59=mozzarella cheese, 64=Parmesan cheese, 66=Pecorino cheese, 76=ricotta cheese, 87=egg, 92=gluten

**Fig. S2:**
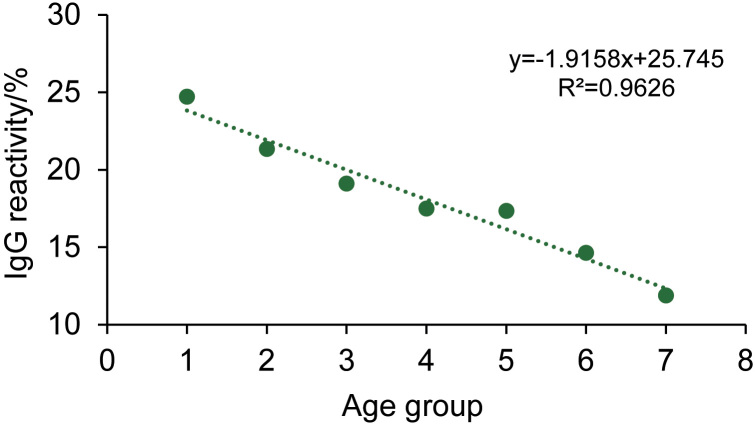
The linear regression of the mean values of the IgG reactivity for the seven age groups (from 0–18 years=group 1 to >70 years=group 7) (see Table S2) is shown with the corresponding equation and R^2^ value

We also analysed the IgG response to the reactive food antigens greater than 20 % depending on the age of the cohort of 6575 subjects (Fig. 4). Seven subgroups were formed; the first included subjects younger than 18 years, five groups covered intervals of about 10 years, and the last comprised subjects older than 70 years. All 14 highly reactive food antigens showed a decrease in the IgG response with increasing age. Considering the mean values derived from the sum of the IgG reactivity percentages for the 14 highly reactive foods reported in Fig. 4 (Table S2), a linear decrease in total reactivity was observed (Fig. S2). Significant differences with p<0.05 were found for the groups of older than 30 compared to the group aged 0-18 (Table S2).

**Fig. 5:**
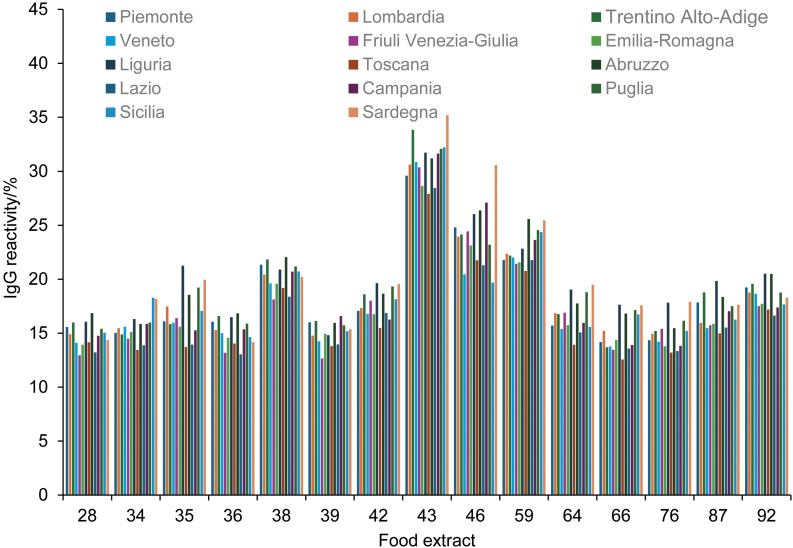
IgG reactivity of 15 highly reactive (>15 %) food antigens determined in 14 Italian regions. 28=Emmer cheese, 34=gorgonzola cheese, 35=Grana Padano cheese, 36=durum wheat, 38=wheat, 39=kamut, 42=goat milk, 43=cow milk, 46=beer’s yeast, 59=mozzarella cheese, 64=Parmesan cheese, 66=Pecorino cheese, 76=ricotta cheese, 87=egg, 92=gluten

We also evaluated the trend of highly reactive food antigens in different Italian regions (Fig. 5). In general, apart from small variations, no significant differences were observed and food reactivity was found independent from the different regions and their specific eating habits.

ELISA, due to its accuracy, specificity and sensitivity, is a versatile immunological analysis method belonging to the category of immunoenzymatic tests (*19*, *20*). In this type of analytical assay, a substance to be measured, defined as an analyte, binds to another, typically an antibody, which detects its presence. The ELISA assay is designed to detect and identify, both qualitatively and quantitatively, a specific substance within a sample, usually in biological matrices. For these reasons, ELISA is the only scientifically proven test for the determination of plasma or serum IgG (and immunoglobulins belonging to other classes such as IgE) titres in healthy and pathological subjects. ELISA tests are generally performed on plastic supports called 96-well plates, which require large quantities of samples and reagents, lengthy analysis times and allow the analysis of only one sample at a time. In contrast, we have developed a microarray system consisting of chips able of analysing 16 samples simultaneously and quantitatively determining the IgG reactivity to 92 food antigens per sample. Moreover, thanks to the properties of microarrays that are similar to those of nanomaterials (*17*-*19*), the present system enables a single operator to manage a high number of samples using small quantities of blood, offering considerable benefits for the patient due to its limited invasiveness, high repeatability of the analysis compared to traditional 96-well plates, reduced consumption of materials (such as plates, plastic material, tips, *etc*.) and minimum use of space (such as warehouse, fridge, workstation, *etc*.). Finally, it shows a remarkable sensitivity and precision based on the use of automation in both chip preparation and its analysis. This is possible because the automation is compact and small, as it operates on very small supports.

According to a previous study (*20*), the quality of food antigen extracts is essential for an accurate IgG reactivity evaluation (*23*) as they have several limitations mainly related to the extraction processes, which may result in the loss of some allergenic proteins, acquisition of proteins from unknown sources, and different protein concentration and composition between production batches. This can lead to the absence or low concentration of proteins in the extracts causing false negative results during diagnosis and ineffectiveness of subsequent therapy. With this in mind, food antigens used to produce the present microarrays are tested for protein content and quality using mono-dimensional SDS-PAGE electrophoresis (*20*) to ensure accurate quality control throughout the purification process and batch-to-batch reproducibility. Furthermore, each food production was tested for biological activity and IgG reactivity using specific serum samples reactive to that specific antigenic extract.

Comparing IgG reactivity in 6880 subjects from the Italian population tested by classic ELISA test on 96-well support in the previous study (*20*) and in this research using microarray assay, we observed a quite similar behaviour regarding highly reactive food antigens. Milk and several milk derivatives, egg, wheat and yeast all produced an IgG response greater than 15-20 % with both technologies. Wheat showed a reactivity between 15 and 20 % and rye an IgG response between 10 and 15 % by ELISA on the 96-well support (*20*), while kamut and gluten were not tested in the previous study. In general, using microarray technology applied to immune-enzymatic test for serum IgG quantification, we observed strong reactivity to proteins belonging to well-known allergenic food antigens (*24*, *25*). Notably, the high reactivity of gluten extract, 18.7 % in the total population, with 18.1 % in males and 19.3 % in females, showed that a large part of Italian population potentially suffers from non-coeliac gluten sensitivity. Patients with this disorder exhibit symptoms similar to coeliac disease and benefit from a gluten-free diet, although medical tests exclude the presence of coeliac disease or wheat allergy (*26*). This high reactivity to gluten was also reported by Ryu *et al.* (*25*) in 30 Korean subjects. According to the previous study (*20*), pineapple, banana, peanut and mushrooms were moderately reactive percentages between 5 and 10 %.

We also confirm that no significant differences were observed between male and female groups. However, contrary to the previous study (*20*), we measured a general decrease in IgG response to highly reactive food antigens with increasing age. This discrepancy may be due to the different selection of groups. In the first study, only two groups with a time span of 40 years were selected, while in this latest research, we selected seven groups with a smaller age range, making the results more reliable. It is possible to hypothesize that subjects with high levels of plasma IgG also experience symptoms of discomfort and various disorders and therefore, on the advice of professionals, tend to reduce consumption of foods that cause the onset of these symptoms (*10*, *27*). Consequently, the plasma IgG content decreases.

More frequently consumed foods have been found to increase IgG compared with less frequently eaten foods, and serum IgG amount for immunogenic food groups is higher than for non-immunogenic groups (*24*). IgG-mediated reactivity against food antigens can arise from their repeated exposure, which may increase the probability of food-specific antigens coming in contact with their antigen-specific T and/or B cells in an inflammatory environment (*27*). This process subsequently activates and induces proliferation of cells similar to those implicated in the generation of autoantibodies against self-tissue and immune diseases (*28*-*30*). It is now clear that the increase in specific plasmatic IgG is a defence mechanism against antigens (non-self molecules) that can cause inflammatory pathologies and in particular allergies (*31*-*33*). In fact, antigens can be ingested due to damage of the intestinal mucosa or due to binding at the level of intestinal cells between antigens and mast cells or basophilic granulocytes present in the lymphatic system, or with receptors exposed on these cells. It is estimated that approx. 15 % of ingested proteins are not fully digested (*32*). The increase in IgG allows to bind the antigen, and this complex in turn binds with mast cells and basophilic granulocytes on a receptor antagonistic to the receptor that binds IgE, stimulating the allergic response (*31*-*33*). This receptor is known as FCγRIIb, and its binding with antigens reduces the response of both mast cells and basophilic granulocytes by reducing the activation of TH_2_ leukocytes with lower production of inflammatory cytokines, histamine, heparin, *etc*. (*32*). This is a defence mechanism against particularly dangerous molecules, known as tolerance. This mechanism prevents the development of allergies but also produces stress in the organism, with excess production of IgG and formation of cell-IgG-antigen complexes that can deposit in tissues and organs, causing inflammation and tissue damage (*27*). There is a balance between IgG and IgE concentrations that can be altered by increased antigen intake and production of IgG immunoglobulins. We should also consider that the blood concentration of IgG required to neutralise antigens able to develop allergies is 100 times greater than that of plasma IgE (10 mg/mL of IgG *vs* 0.1 mg/mL of plasma IgE), potentially leading to a significant accumulation of this class of immunoglobulin (*32*). With increased antigen intake and continuous exposure, particularly to highly allergenic foods, activation and proliferation of all cells (mast cells, basophilic granulocytes, and T and B lymphocytes) results in loss of tolerance with a mechanism similar to the production of autoantibodies, autoimmune diseases and inflammation (*27*). These autoantibodies can also target components of the junctions of the intestinal epithelium, such as occludin, leading to increased intestinal permeability (*27*) and the development of food allergies with production of IgE, which has been shown to be produced more in situations of increased intestinal permeability (*27*, *34*). Several findings suggest that the presence of IgG reactive to food antigens, rather than the presence of symptoms, is perhaps more indicative of a simultaneous increase in intestinal permeability, and that in some cases these IgG-mediated reactions to food antigens reach clinical significance, producing symptoms, while in other cases they do not (*27*, *35*). Consequently, a gastrointestinal symptom status of an individual, *e.g*. symptomatic *vs* asymptomatic, should not exclude the possibility of increased permeability, as growing evidence highlights the connection between increased intestinal permeability and many chronic diseases, which may have prevention-oriented implications in clinical practice. Clinicians recommending food IgG assays should also consider the increased permeability that may be present in patients who tested positive for food IgG, and sensitivity to food antigens should be considered during clinical evaluation as a potential cause but also as a consequence of increased intestinal permeability (*27*).

Plasma IgG testing is clearly not indicated for the diagnosis of food allergies (*30*) and although its clinical implications remain under discussion (*36*), according to previous considerations, IgG determination against food antigens has several scientifically proven benefits. In particular: (*i*) it allows evaluation of a potentially harmful state for the organism at a preclinical level without obvious symptoms, (*ii*) it highlights a highly probable potential inflammatory state of the intestinal mucosa, including implications of alteration of the microbiota and therefore dysbiosis, (*iii*) it helps prevent and limit the state from evolving towards a highly inflammatory and allergic condition with IgE production and serious symptoms, and (*iv*) it reduces a preclinical and subchronic inflammatory state. This is confirmed by several studies showing that elimination of foods based on IgG food sensitivity test results also reduces gastrointestinal symptoms associated with various diseases (*17*, *37*-*39*). Furthermore, it is now well known that food allergy is not caused solely by the IgG4 subclass, but it is also by other IgG subclasses, all of which are known to have measurable affinity for FcγRIIb. Consequently, only the assay of all IgG classes can give correct information (*32*).

In a recent paper (*25*), a microarray was fabricated by immobilising 66 food antigens on activated glass slides, and serum IgG of 30 Korean subjects was analysed also in relation to their dietary patterns. According to the authors, the developed microarray showed very good performance and potential for use as an automated assay system. Moreover, the authors observed that immunogenic and most consumed foods, such as egg, milk and gluten, induced relatively high immune responses. These results, obtained in Korean subjects with food antigens typical of that population, agree with our results, even though ours were obtained using antigens typical of the Italian population. Finally, in the same study (*25*), the authors demonstrated that the use of serum or directly tested whole blood produced comparable results. This also agrees with our procedures, in which we used dried blood instead of serum or plasma in our microarray technology, obtaining similar results (see also reference (*21*)).

## CONCLUSIONS

The microarray test used in this study is a tool for identifying and measuring amounts of specific IgG antibodies in the blood. It can reveal triggers of intolerance and help the doctor or nutritionist choose the best treatment for the patient. Microarray is a robust method with good reproducibility and low variation, as demonstrated in this validation study. Moreover, microarray technology enables high throughput, ensuring that a large number of samples can be analysed with significant savings in time, reagents and costs.

## SUPPLEMENTARY MATERIAL

Supplementary material is available at https://www.ftb.com.hr/images/pdfarticles/2026/April-June/FTB-64-196-S1.pdf.

## Appendix

**Table S1:** The list of 92 food items from which food protein antigens were prepared

Food extract	Label	Food extract	Label	Food extract	Label	Food extract	Label
Apricot	1	Coconut	24	Lemon	47	Peas	70
Amaranth	2	Rabbit	25	Pork	48	Chicken	71
Cashew nut	3	Green beans	26	Corn	49	Tomato	72
Pineapple	4	Bean	27	Mandarin	50	Grapefruit	73
Peanut	5	Emmer	28	Almond	51	Prune	74
Orange	6	Broad bean	29	Beef	52	Quinoa	75
Asparagus	7	Fennel	30	Apple	53	Ricotta	76
Oat	8	Strawberry	31	Eggplant	54	Rice	77
Avocado	9	Champignon mushroom	32	Melon	55	Salmon	78
Salted cod	10	Shrimp	33	Cod	56	Rye	79
Banana	11	Gorgonzola	34	Honey	57	Mackerel	80
Chard	12	Grana Padano	35	Blueberry	58	Sole	81
European bass	13	Durum wheat	36	Mozzarella cheese	59	Soy	82
Broccoli	14	Buckwheat	37	Hazelnut	60	Spinach	83
Cocoa	15	Wheat	38	Walnut	61	Turkey	84
Coffee	16	Kamut	39	Gilthead bream	62	Tuna	85
Artichoke	17	Kiwi	40	Barley	63	Trout	86
Carrot	18	Raspberry	41	Parmesan cheese	64	Egg	87
Cauliflower	19	Goat’s milk	42	Potato	65	Cabbage	88
Chickpea	20	Cow’s milk	43	Pecorino cheese	66	Veal	89
Cucumber	21	Lettuce	44	Peppers	67	Pumpkin	90
Chicory	22	Lentils	45	Pear	68	Zucchini	91
Onion	23	Brewer's yeast	46	Fishing	69	Gluten	92

**Table S2:** The mean values derived from the sum of the IgG reactivity for the 14 highly reactive foods shown in Fig. 3 are presented according to the age group. The coefficient variation (CV) and the significance of differences compared to the 0–18 age group is also shown. The linear regression of the mean values of the IgG reactivity for the seven age groups (from 0–18 years=group 1 to >70 years=group 7) is also presented, with the corresponding equation and R^2^ value given in Fig. S2

	Age group/year						
Parameter	0–18	18–29	30–39	40–49	50–59	60–69	>70
Mean value	24.7	21.4	19.1	17.5	17.4	14.6	11.9
CV/%	23.6	24.4	26.8	26.9	26.4	24.6	32.2
p-value		0.80639	0.03660	0.00193	0.00164	0.00001	0.00000
